# Survey on *Sarcocystis* in bovine carcasses slaughtered at the municipal abattoir of El-Kharga, Egypt

**DOI:** 10.14202/vetworld.2016.1461-1465

**Published:** 2016-12-21

**Authors:** Ali Meawad Ahmed, Nagwa Thabet Elshraway, Ahmed Ibrahim Youssef

**Affiliations:** 1 Department of Food Hygiene, Faculty of Veterinary Medicine, Suez Canal University, Ismailia, Egypt; 2 Department of Food Hygiene, Faculty of Veterinary Medicine, Assuit University, New Valley, Egypt; 3 Department of Animal Hygiene and Zoonoses, Division of Zoonoses, Faculty of Veterinary Medicine, Suez Canal University, Ismailia, Egypt

**Keywords:** bovine, meat, *Sarcocystis fusiformis*, *Sarcocystis hirsuta*, *Sarcocystis*

## Abstract

**Aim:**

The main objectives of this study were to determine the incidence of *Sarcocystis* sp. infection in cattle and buffalo carcasses slaughtered at El-Kharga abattoir, New Valley Governorate, Egypt.

**Materials and Methods:**

The slaughtered animals were daily inspected for *Sarcocystis* macrocysts through a year (2015). Macroscopic *Sarcocystis* was detected from a total of 2120 cattle and buffalo carcasses. In addition, 100 meat samples were collected from female cattle and buffalo (50 each) and were examined microscopically for sarcocystosis.

**Results:**

The overall incidence of *Sarcocystis* macrocyst among bovine carcasses was 159/2120 (7.5%). Total incidence in cattle was 149/2000 (7.45%), whereas it was 10/120 (8.33%) in buffalo carcasses. Concerning gender, the overall prevalence of *Sarcocystis* infection was 127/1790 (7.09%) in male and 32/330 (9.69%) in females bovine carcasses. The highest detection rate of *Sarcocystis* lesions was from the esophagus (76.3%) followed by throat muscles (35.3%), tongue (33.8%), and diaphragm muscles (18.71%). Macrocysts from cattle were identified to *Sarcocystis hirsuta*, whereas *Sarcocystis fusiformis* was identified from buffalo carcasses. By microscopic examination, 18 (36%) of 50 female cattle carcasses harbor *Sarcocystis* sp., whereas 11 (22%) of buffalo carcasses were harbored *Sarcocystis* microcysts.

**Conclusion:**

A high incidence of *Sarcocystis* infection was detected among slaughtered bovines in El-Kharga abattoir, Egypt. *Sarcocystis* macrocysts were a higher incidence in female elder animals macrocysts were identified to *S. hirsuta* in cattle and *S. fusiformis* in buffaloes. Sarcocystosis constitute a major cause of economic losses at El-Kharga abattoir. Beef meat may carry health risks to consumers.

## Introduction

*Sarcocystis* is an intracellular protozoan parasite belongs to the phylum Apicomplexa and family Sarcocystidae. It is cyst-forming intracellular coccidian parasites with obligate two hosts. *Sarcocystis* needs two obligatory hosts during its life cycle, including a carnivorous as a definitive host and an omnivorous or herbivorous as an intermediate host [1]. There are many economic impacts of sarcocystosis. The pathogenic species affect cattle may lead to severe, fatal disease leading to abortion, reduced milk yield, neurologic signs, and loss of weight. The infection with macroscopic *Sarcocystis* cysts renders the meat unmarketable and leads to downgrading and condemnation of the carcasses [2,3].

Sarcocystosis is commonly seen in domestic animals such as buffaloes, cattle, and pigs. Meat and meat products are the main sources of infection to the human beings through ingestion of well-developed tissue cysts containing bradyzoites [4]. Zoonotic species, *Sarcocystis hominis* and *Sarcocystis suihominis* can cause digestive disturbances such as nausea, vomiting, diarrhea, and other gastrointestinal symptoms in infected patients [5]. *Sarcocystis* can cause intestinal and muscular sarcocystosis in human persistent myalgia, episodic weakness, subcutaneous nodules and dermatomyositis, if they consumed a raw or inadequately cooked beef infected with sporocysts [6-8].

In Egypt, high incidences of *Sarcocystis* among bovine carcasse*s* have been recorded with great variations among species and localities [9,10]. Two species, *Sarcocystis fusiformis* and *Sarcocystis levinei*, were commonly detected in buffaloes in different localities in Egypt [7-10]. In addition, the microscopic zoonotic *S. hominis* cysts were commonly detected in many studies [11,12].

To our knowledge, this is the first study on sarcocystosis conducted in the New Valley Governorate. Therefore, the objectives of this study were to determine the incidence of *Sarcocystis* affecting slaughtered cattle and buffalo in the municipal abattoir of El-Kharga, Egypt.

## Materials and Methods

### Ethical approval

This study has been approved by the Animal Rights and Ethical Use Committee of Suez Canal and Assiut University.

### The study area

El-Kharga city is the capital of New Valley Governorate. It is a part of the oasis which is located to the west of the Nile Valley. New Valley Governorate is located 232 km to the South of Cairo and represented it is about 45% from the total Egypt area.

El-Kharga abattoir slaughtered annually from 2000 to 3000 bovine animals. According to the Egyptian legislations of meat inspection, male bovines aged more than 2 years are only approved from slaughtered. It is forbidden to slaughter female bovines except after all teeth are changed (over 5 years).

### Samples collection

A total of 2000 local breed cattle and 120 local breed buffaloes, slaughtered at El-Kharga abattoir, were daily inspected for the presence of *Sarcocystis* macrocyst for one the year 2015. Esophagus, throat muscles, tongue, diaphragm, and heart were efficiently inspected by naked eye and palpation for the presence of macrocysts. 100 g of the infected tissue or organs were collected in clean plastic bags. The carcasses under investigation were assigned into two groups: The first one was males aged from 2 to 3 years including 1700 steer and 90 buffaloes. The second group was females over 5 years of age including 300 cows and 30 buffaloes. All data were recorded, and all samples were transported in an icebox to the laboratory for further examinations and species identification.

### Macroscopic identification

*Sarcocystis* was identified by visual inspection of the muscular tissues for detection of *Sarcocystis* cysts according to Huong [10]. The revealed cysts were dissected out of the tissue and measured by a transparent plastic ruler.

### Microscopic examination of muscle tissues

Microscopic *Sarcocystis* cysts were performed using muscle squeeze method following the protocol [3]. 1 g of fresh muscles was cut into small pieces, approximately 3-5 mm thick, and crushed strongly between two glass slides and after staining with Giemsa examined under the microscope (400×).

### Statistical analysis

GraphPad Instant version 3 was used for determination of means and the analysis of variance between the different data. The treatment, in this study, was determined using standard error and analysis of variance (p<0.05).

## Results

### The incidences of *Sarcocystis* infection to the bovine carcasses

As tabulated in Table-1, results revealed that the overall incidence of *Sarcocystis* macrocyst among bovine carcasses was 159/2120 (7.5%). Total incidence in cattle was 149/2000 (7.45%), whereas it was 10/120 (8.33%) in buffalo carcasses. The highest incidence was reported in female buffaloes, 3/30 (10%) and female cattle 29/300 (9.67%). The lowest incidence was recorded in male cattle and male buffalo as 120/1700 (7.06%) and 7/90 (7.78%), respectively.

**Table-1:** Incidence of *Sarcocystis* in cattle and buffalo carcasses slaughtered in El-Kharga Abattoir.

Animal species	Animal gender				Total	

	Females		Males					
		
	Inspected N	Infected N (%)	Inspected N	Infected N (%)	Inspected N	Infected N (%)
Cattle	300	29 (9.67)	1700	120 (7.06)	2000	149 (7.45)
Buffaloes	30	3 (10)	90	7 (7.78)	120	10 (8.33)
Total	330	32 (9.69)	1790	127 (7.09)	2120	159 (7.5)

### The monthly incidence of *Sarcocystis* macrocysts in bovine slaughtered at El-Kharga abattoir

**Figure-1:**
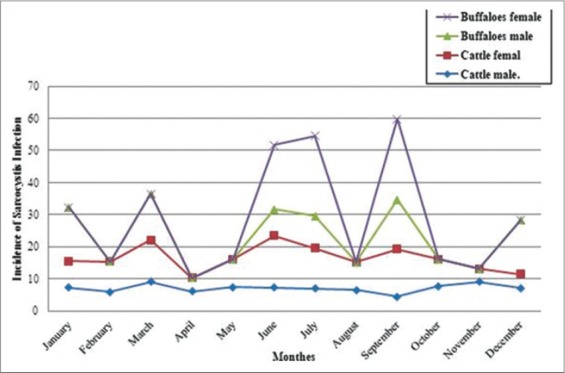
Monthly incidence of sarcocystis infection in male and female slaughtered cattle and buffaloes in El-Kharga abattoir.

As illustrated in Figure-1, the results revealed that the monthly incidence of *Sarcocystis* macrocysts in male and female cattle and buffaloes at El-Kharga abattoir from January to December 2015 for cattle ranged from 4% (in April) to 16.13% (in June) with relatively regular pattern in females which was higher incidences than males. In buffalos, a monthly distribution of the *Sarcocystis* macrocyst incidences showed an irregular pattern. *Sarcocystis* macrocysts were detected only in January, March, June, July, September and December by incidences of (16.67%), (14.29%), (8.33%), (10%), (15.38%) and (16.67%), respectively in male buffalo. However, the macrocysts in female buffalo were detected only on June, July, and September as 1 (20%), 1 (25%), and 1 (25%), respectively.

### Organ distribution of the *Sarcocystis* macrocysts

Regarding the organ distribution of the *Sarcocystis* macrocysts, results tabulated in Table-2 revealed that the highest detection rate of *Sarcocystis* lesions was found esophagus 10^6^ (76.26%), followed by throat muscles 49 (35.25%), tongue 44 (33.81%), and 26 (18.71%). No *Sarcocystis* infection was detected in heart muscles.

**Table-2:** *Sarcocystis* infection in different organs of slaughtered cattle and Buffalo.

Organs	N (%)				Total N (%)

	Cattle		Buffaloes		
	
	Males	Females	Males	Females	
Esophagus	90 (5.29)	8 (2.67)	5 (5.56)	3 (10.0)	106^a^ (76.26)
Throat muscles	45 (2.65)	2 (0.67)	1 (11.1)	1 (3.33)	49^b^ (35.25)
Tongue	34 (2.00)	7 (2.33)	2 (2.22)	1 (3.33)	44^c^ (33.81)
Diaphragm	20 (1.18)	5 (1.67)	1 (1.11)	-	26^d^ (18.71)
Heart	-	-	-	-	-

a, b, c, d Means followed by a different letter in the line are significantly different (p>0.05) T. muscles=Throat muscles (neck muscles)

### *Sarcocystis* sp. identification based on macroscopic examination

**Figure-2:**
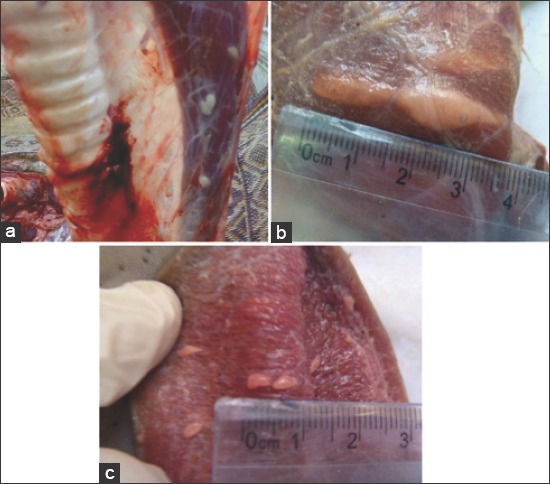
Macroscopic Sarcocyts detected from bovine carcasses. (a) Large sized sarcocystis observed in esophagus female buffaloes at El-kharga abattoir. (b) Large sized Sarcocysts (0.5 cm) observed in Oesophagus (1.5 cm) at El-kharga abattoir. (c) Large sized Sarcocysts (0.5 cm) observed in female tongue buffaloes at El-kharga abattoir.

Regarding *Sarcocystis* sp. identification, macroscopic examination revealed that macrocysts from buffalo were identified to *S. fusiformis* sp. which were fusiform or spindle-shaped, white or creamy color, ranged from 1.0 to 7.23 × 1.0 to 1.5 mm (4.63 mm × 1.25 mm) (Figure-2a). However, macrocysts from cattle were identified to *Sarcocystis hirsuta* where the shape of the *Sarcocystis* was oval, the size about 114 µm × 50.81 µm cyst size and wall thickness of 1.11 µm (Figure-2b and c).

Microscopically, Out of 50 female cattle carcasses, 18 (36%) harbor *Sarcocystis* sp., whereas, out of 50 female buffalo carcasses, 11 (22%) harbored *Sarcocystis* microcysts.

## Discussion

*Sarcocystis* sp. is normally developed in two host cycles consisting of the intermediate host (prey) and the final host (predator). Each host may be infected with more than one *Sarcocystis* sp. [13]. Cats and dogs are dropping many sporocysts in the external environment for the lengthy duration of each meal of infected meat. The main source of infection for cattle and buffalo was the contaminated feed and water by the infective stage of *Sarcocystis* sp. Life cycles in cattle sarcocystosis have been established for cattle-dog (*Sarcocystis cruzi*), cattle-cat (*S. hirsuta*), cattle-human (*S. hominis*), and others [6]. The New Valley governorate is located in a desert area where stray carnivores such as dogs and cats are abundant. The widespread existence of sporocysts in the environment is attributed to the abundance of definitive host, and the resistance of sporocyst to harsh environmental conditions [13]. This suggests that bovines are frequently exposed to infection due to their close relationship with dogs, cats, and even wild animals that act as final hosts for these protozoa.

In this study, the overall incidence of *Sarcocystis* macrocyst among bovine carcasses was come in agrees with that obtained by El-Dakhly *et al*. [14], who reported (6.9%). However, another study revealed 3% incidence in Egypt [15]. On the other hand, higher incidences of sarcocystosis have been recorded in Egypt, reaching 94% [11], 52% [16], and 100% [17,18]. In addition, higher infection rates have been recorded in other countries that have similar climatic conditions; 87% in India [12], 71.5% from beef cattle in Egypt [19], (29%) [20], 95% [21] 82.9% in Iraq [22], 65% in the Philippine [23], and 57% in Iran [24]. The lower prevalence of *Sarcocystis* macrocysts among slaughtered cattle and buffalo at El-Kharga abattoir could be interpreted as the unfavorable climate condition for the survival of cysts in the New Valley governorate, Egypt. The New Valley area characterizes by a very arid climate which is not favored for the survival of *Sarcocystis* in this environment.

Our results revealed that *Sarcocystis* macrocysts were the most common in cattle than buffalo and the infection rate increased by ages. This finding is most likely due to longer exposure periods of aged animals to the sporocysts infection. The frequent contact between ruminant animal and final hosts is allowing life cycle of the protozoan and spread of infection. By increasing in the slaughter the age of the animal, animal repeated exposure to infestations, which gradually accumulate cysts in muscle [25]. In addition, the cysts needed longer time to appear macroscopically compared to microscopic cysts [14,21]. Moreover, the lower number of the examined aged female bovine carcasses matched with younger steer could affect the infection rate.

The monthly incidence of *Sarcocystis* macrocysts in bovine animals slaughtered at El-Kharga abattoir from January to December for cattle revealed that there were no significant seasonal effects on the incidences of *Sarcocystis*. Sarcocystis incidences were relatively higher in hot seasons. This finding might be attributed to the longer grazing periods during the summer season. This finding was supported by the finding of a previous study that showed that the highest incidence value for female buffaloes reported in January and September, while the highest incidence value for male buffaloes reported in July and September [10].

Regarding the organs distribution of the *Sarcocystis* macrocysts, the results revealed that the highest detection rate of *Sarcocystis* lesions was found in the esophagus (76.26%) followed by throat muscles (35.25%), tongue (33.81%), and (18.71%). The highest infection rate in esophagus was consistent with the previous studies [3,7,15,17,26]. In contrary, in a surveillance about the prevalence of sarcocystosis in cattle and water buffaloes in peninsular Malaysia, Sarcocystis was predominant in the skeletal muscles and diaphragm (27% each), and heart (66.7%), in cattle and water buffaloes, respectively [8]. However, Daryani *et al*. [24] found that the abdominal muscles of infected buffaloes were more frequently infected than the esophagus.

In this study, macroscopic examination revealed that macrocysts were identified to *S. fusiformis* sp. in buffalo and *S. hirsuta* in cattle. The detection of *S. fusiformis* was consistent with the previous reports from Egypt [9,21]. In this study, high incidences of microscopic sarcocystosis might include *S. hominis*. Microscopically, out of 50 female cattle carcasses, 18 (36%) harbor *Sarcocystis* sp., whereas 11 (22%) out of 50 buffalo harbored *Sarcocystis* microcysts. It has been reported that the consumption of improperly cooked infected meat with *Sarcocystis* is the main source of transmission of the infection to human [10,16,25,26]. Humans could act as intermediate hosts after eating inadequately cooked beef, a patient suffering from abdominal discomfort and loose stools [21].

## Conclusion

The results of this work indicated a high incidence of *Sarcocystis* infection among slaughtered bovines in El-Kharga abattoir, Egypt. Sarcocystis macrocysts were higher incidence in elder animals especially in females than males. Macrocysts were identified to *S. hirsuta* in cattle and *S. fusiformis* in buffaloes. Bovines slaughtered at El-Kharga abattoir may carry health risks to consumers from *Sarcocystis* lesions. Therefore, the concerned authority should make efforts for proper meat inspection procedures and combating street dogs and cats.

## Authors’ Contributions

AMA: Study design, photography and revision of the manuscript. NTE: Study design, collection of the samples, photography, collection of data from the slaughter house, drafted and revised the manuscript. AIY: Corresponding author of the manuscript, drafted and revised the manuscript, helped in laboratory examination and data analysis. All the authors shared laboratory examination and data analysis. All authors have read and approved the final manuscript.
